# A polymer-direct-intercalation strategy for MoS_2_/carbon-derived heteroaerogels with ultrahigh pseudocapacitance

**DOI:** 10.1038/s41467-019-09384-7

**Published:** 2019-03-26

**Authors:** Nan Feng, Ruijin Meng, Lianhai Zu, Yutong Feng, Chengxin Peng, Jimei Huang, Guanglei Liu, Bingjie Chen, Jinhu Yang

**Affiliations:** 1 School of Chemical Science and Engineering, Tongji University, Shanghai, 200092 China; 2 Research Center for Translational Medicine and Key Laboratory of Arrhythmias of the Ministry of Education of China, East Hospital Tongji University School of Medicine, No. 150 Jimo Road, Shanghai, 200120 China; 3 School of Materials Science and Engineering, University of Shanghai for Science and Technology, Shanghai, 200093 China

## Abstract

The intercalation strategy has become crucial for 2D layered materials to achieve desirable properties, however, the intercalated guests are often limited to metal ions or small molecules. Here, we develop a simple, mild and efficient polymer-direct-intercalation strategy that different polymers (polyethyleneimine and polyethylene glycol) can directly intercalate into the MoS_2_ interlayers, forming MoS^2^-polymer composites and interlayer-expanded MoS_2_/carbon heteroaerogels after carbonization. The polymer-direct-intercalation behavior has been investigated by substantial characterizations and molecular dynamic calculations. The resulting composite heteroaerogels possess 3D conductive MoS_2_/C frameworks, expanded MoS_2_ interlayers (0.98 nm), high MoS_2_ contents (up to 74%) and high Mo valence (+6), beneficial to fast and stable charge transport and enhanced pseudocapacitive energy storage. Consequently, the typical MoS_2_/N-doped carbon heteroaerogels exhibit outstanding supercapacitor performance, such as ultrahigh capacitance, remarkable rate capability and excellent cycling stability. This study offers a new intercalation strategy which may be generally applicable to 2D materials for promising energy applications.

## Abstract (web summary)

Methods to fabricate layered materials are often associated with harsh conditions and complicated manipulations. Here the authors report a polymer-direct-intercalation strategy to synthesize composite heteroaerogels consisting of molybdenum sulfide/carbon nanosheets for high-capacitance supercapacitors.

## Introduction

To meet ever-increasing energy demands, the exploration of advanced electrode materials has been triggered for developing state-of-the-art energy storage devices. Among various energy storage devices, supercapacitors are promising due to ultrahigh power output and ultralong operating lifetime^1^. There are two different working mechanisms in supercapacitors, including electrochemical double layer capacitive (EDLC) energy storage^2,3^ and pseudocapacitive (PC) energy storage^4^. The first mechanism is realized through physically adsorbing opposing electrolyte charges in two electrodes, featuring high power and long cycling life, while the latter relates to fast and reversible redox reactions occurring over electrodes, significantly elevating the capacitance and meanwhile maintaining high rate capability. The mechanisms are largely determined by the electrode materials employed and play a decisive role in the performance of supercapacitors. When the two mechanisms work together in a supercapacitor, a high comprehensive performance is expected due to their synergistic effect. However, supercapacitors are still suffering from low energy densities, which is an obstacle for their practical applications. Current efforts mainly focus on the development of high-capacitance supercapacitors with simultaneous high power. To achieve high specific capacitance, electrode materials should be designed with high porosity, good electrical conductivity, and especially, feasible redox reactions for efficient PC energy storage.

**Fig. 1:**
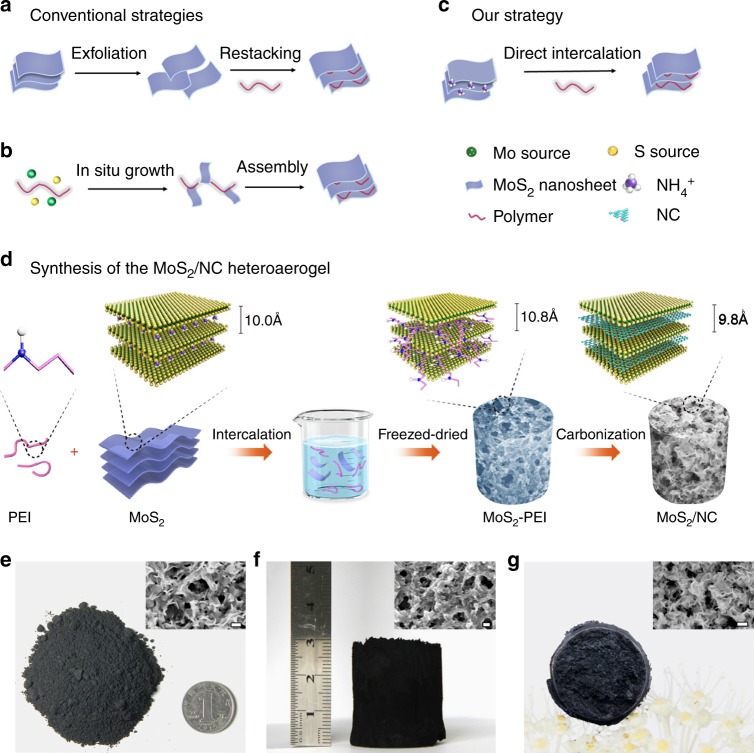
Comparison of different strategies and the polymer direct intercalation. **a**–**c** The schematic illustrations show the conventional synthetic strategies to realize interlayer-expended MoS_2_ (**a**, **b**) and the polymer-direct-intercalation (PDI) strategy for the synthesis of a MoS_2_/NC heteroaerogel (**c**) to prepare interlayer-expended MoS_2_/carbon composites. **d** The schematic illustration for the synthetic procedure of the MoS_2_/NC heteroaerogel. **e**–**g** Optical photographs of the as-synthesized pre-intercalated MoS_2_ nanosheets (**e**), the MoS_2_-PEI (PEI, Mw = 600) composite after freeze-drying (**f**), and the MoS_2_/NC heteroaerogel after carbonization (**g**). Insets of (**e**–**g**) show the corresponding SEM images with scale bars of 100 nm

Recently, MoS_2_ nanosheets, an analogue of graphene with a two-dimensional layered nanostructure, have attracted tremendous attention for supercapacitors due to their unique structural and electronic properties^5–7^. Specifically, MoS_2_ nanosheets are stacked by S–Mo–S monolayers via weak van der Waals forces along the *c* axis, which allows easy intercalation of foreign ions (H^+^, K^+^, NH_4_^+^) for interlayer charge storage^8^. In addition, the central Mo ions hold a range of oxidation states from +2 to +6, exhibiting great potential in high-capacitance PC storage^9^. However, the performance of MoS_2_-based supercapacitors is still limited by the poor electrical conductivity and few accessible active sites of MoS_2_. Considerable efforts have been made to either improve the conductivity of MoS_2_ by integrating conductive carbon materials (graphene^10–13^, carbon nanotubes^14–17^, and conducting polymers^18–20^), or expose more active sites of MoS_2_ nanosheets through intercalation using small molecules or ions. In particular, further strategies combining the above mentioned two solutions to simultaneously increase conductivity and extended interlayer of MoS_2_ have also been developed for the preparation of MoS_2_/carbon composites^21,22^. Conventionally, the synthesis strategies can be categorized as “top-down” and “bottom-up” by employing polymers as both carbon sources and intercalation agents, as depicted in Fig. 1a, b. The “top-down” strategy involves the first exfoliation of existing MoS_2_ by intercalation of small molecules or ions, followed by the mixing and restacking of MoS_2_ monolayers and polymers, which often accompanies with the formation of highly unstable and flammable intermediates^15,23–27^. The “bottom-up” strategy was realized through the in situ nucleation and growth of MoS_2_ monolayers on polymer molecules (polyaniline, polyvinyl pyrrolidone, polyethylene oxide, etc.), which requires precise control of polymer-mediated crystal growth in thermodynamics and dynamics to avoid the formation of bulk MoS_2_ and phase separation between MoS_2_ and polymers^28–35^. Despite great progress, it is noted that the conventional strategies are often associated with complicated approaches such as extra intercalation manipulation or harsh growth conditions. In addition, the resulting MoS_2_/carbon composites are usually in the form of powders, especially when the MoS_2_ active material is in high content (over 50%) in the composites, which may lead to a high contacting resistance as well as an unstable and ineffective ion diffusion pathway caused by the aggregation of MoS_2_. Therefore, it is highly desirable to develop a new, simple, and efficient strategy for constructing MoS_2_/carbon composites with architectures/nanostructures that are favorable for high-performance supercapacitors.

In this work, we propose a novel polymer-direct-intercalation (PDI) strategy to construct three-dimensional (3D) MoS_2_/N-doped carbon (MoS_2_/NC) composite heteroaerogels assembled by layered MoS_2_/carbon heteronanosheets for ultrahigh-capacitance supercapacitors. The strategy, which is simple, mild, and efficient, is realized at room temperature by direct intercalation of polymer molecules into the interlayers of prepared MoS_2_ nanosheets (Fig. 1c), followed by carbonization treatment. The resulting MoS_2_/NC heteroaerogels possess a 3D conductive N-doped MoS_2_/C framework, an overlapped MoS_2_/C-layered hetero-interface and expanded interlayers of MoS_2_ nanosheets, conducive not only to fast and stable charge transfer/diffusion, but also enhanced ion intercalation pseudocapacitance. Moreover, when employed as electrodes for supercapacitors, the MoS_2_/NC heteroaerogel with a high content of MoS_2_ (up to 74%) and a high valence (+6) of central Mo ions can deliver an ultrahigh specific capacitance of 4144 F g^−1^ at 1 A g^−1^, remarkable rate capability (2483 F g^−1^ at a drastically increased current density of 10 A g^−1^), and excellent cycling stability with a capacitance loss of ~8% after 8000 cycles at 10 A g^−1^.

## Results

### Synthesis of the MoS_2_/N-doped carbon heteroaerogel

Figure 1d shows the typical preparation procedure of the MoS_2_/NC composite aerogel by polymer (polyethyleneimine, PEI, Mw = 600, 1800, 10,000, 70,000) direct intercalation. First, pre-intercalated MoS_2_ nanosheets with interlayer distance of ~10.0 Å were synthesized hydrothermally with the assistance of ammonium and then were mixed with PEI at room temperature for intercalation. It is noted that the pre-intercalated MoS_2_ nanosheets are in the form of powder and can be produced in a large amount. The pre-intercalated MoS_2_ nanosheets are intertwined and stacked into a 3D network (scanning electron microscope (SEM) image, inset in Fig. 1e) with a relatively high specific surface area of ~99.99 m^2^ g^−1^ (Supplementary Fig. 1a). The pre-intercalated MoS_2_ nanosheets were detected to possess a negative charge surface (zeta potential −30.4 mV, Supplementary Fig. 2) in neutral aqueous solution. Therefore, PEI was preferentially selected as the intercalator owing to the strong electrostatic interaction between its positively charged NH_2_^+^ groups and negatively charged MoS_2_ nanosheets. As a result, PEI molecules readily adsorbed on the surface of MoS_2_ nanosheets and inserted into the interlayers of MoS_2_ nanosheets assisted with the ultrasonic treatment. With intercalation, an aerogel-like 3D MoS_2_-PEI (PEI, Mw = 600) composite instead of powder was formed after freeze-drying due to the linkage of PEI molecules between MoS_2_ nanosheets, showing a cylinder-like shape with a diameter of ~2.5 cm and a height of ~3 cm (Fig. 1f). The coating of PEI on MoS_2_ nanosheets was clearly visible (inset of Fig. 1f). Finally, the MoS_2_/NC heteroaerogel with the expended MoS_2_ interlayers was obtained through in situ transformation of the MoS_2_-PEI composite at 800 °C. The shape of the MoS_2_/NC heteroaerogel was well preserved compared with its MoS_2_-PEI precursor, despite slight volume shrinkage (Fig. 1g). The tap density of the MoS_2_/NC heteroaerogel was calculated to be approximately 0.1263 g cm^−3^, which makes the aerogel stand freely on a *Fatsia japonica* flower^36^. Furthermore, thanks to a variety of stacked pores, the MoS_2_/NC heteroaerogel exhibits a 3D porous architecture (inset of Fig. 1g) and possesses a specific surface area of ~120.4 m^2^ g^−1^ (Supplementary Fig. 1b), slightly higher than that of the pure MoS_2_ due possibly to more exposed interlayer surface through the intercalation and the contribution of the carbon component.

### Structure and composition of the MoS_2_/N-doped carbon heteroaerogel

**Fig. 2:**
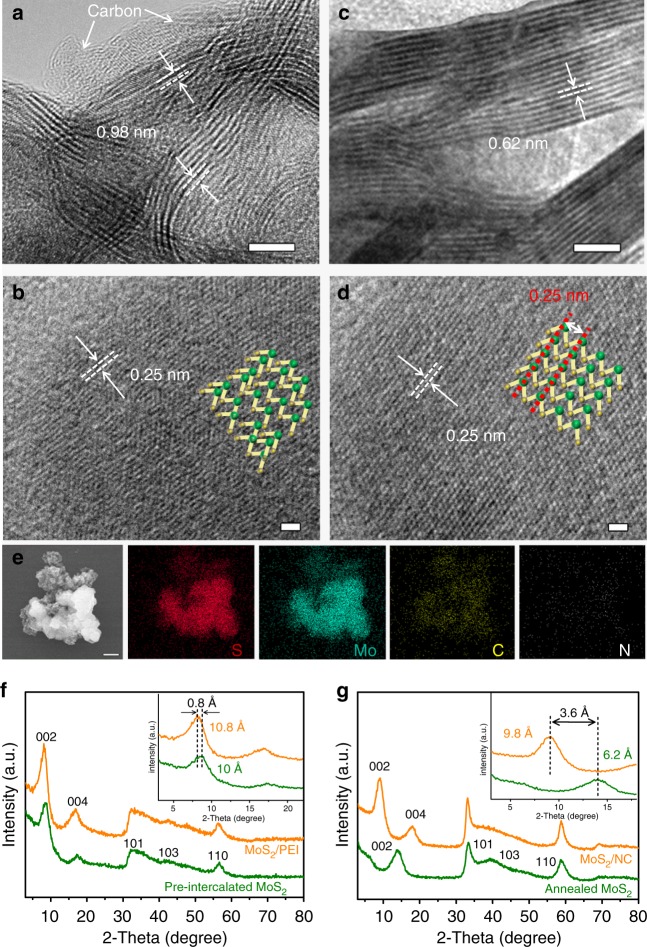
Characterization of the MoS_2_/NC heteroaerogel and MoS_2_ nanosheets. **a**–**d** TEM images of the MoS_2_/NC heteroaerogel derived from the intercalation of PEI (Mw = 600) (**a**, **b**) and the annealed MoS_2_ nanosheets (**c**, **d**). Insets of (**b**, **d**) show the corresponding SAED patterns. **e** SEM-EDS mapping images of the MoS_2_/NC heteroaerogel. **f** XRD patterns of the MoS_2_ nanosheets and MoS_2_-PEI composite obtained after freeze-drying. **g** XRD patterns of MoS_2_/NC heteroaerogel obtained after thermal treatments. Scale bars: **a**, **c** 5 nm; **b**, **d** 1 nm; **e** 1 µm

A high-resolution transmission electron microscopy (HRTEM) image of the MoS_2_/NC heteroaerogel in Fig. 2a shows that MoS_2_ nanosheets are in few layers, showing a tangled and distorted morphology with an expanded interlayer distance of ~0.98 nm, which confirms a graphene-like carbon monolayer inserting into MoS_2_ interlayers^30^. The possible mechanism forming graphene-like carbon monolayers in the MoS_2_ interlayers after carbonization is discussed (Supplementary Note 1). In addition, a thin carbon layer is also observed on the surface of MoS_2_ nanosheets induced by the adsorption and subsequent in situ carbonization of PEI on MoS_2_ nanosheets, indicating the formation of an interconnected network of graphitized carbon throughout the heteroaerogel. It is also found that the crystal lattices in (002) plane of MoS_2_ nanosheets also are distorted to some extent, caused by the introducing foreign atoms (N, C) onto the surface or into interlayers of MoS_2_ nanosheets, as shown in Fig. 2b. Figure 2c shows the TEM image of pristine MoS_2_ nanosheets obtained after thermal treatment. Differently, the MoS_2_ nanosheets show a clean surface, consisting of orderly arranged thin monolayers with a normal displacing of ~0.62 nm for (002) plane^10,37^. In addition, a regular atomic arrangement in (002) plane with the lattice distance of 0.25 nm, corresponding to the (101) plane of MoS_2_^30^ (Fig. 2d). The results above indicate that the graphitized carbon stably exists both in the interlayers and on the surface of MoS_2_ nanosheets through the direct intercalation of PEI. We further employed energy dispersive X-ray spectrometry (EDS) to analyze the chemical composition of the MoS_2_/NC heteroaerogel. EDS data reveals that Mo, S, C, and N elements coexist and distribute uniformly throughout the whole heteroaerogel (Supplementary Figs. 3, 2e), further verifying the successful formation of the MoS_2_/NC heteroaerogel. The above results demonstrate that the direct intercalation strategy is effective, which enables not only the adsorption of PEI molecules onto MoS_2_ nanosheets, but also the intercalation into the interlayers of MoS_2_ nanosheets. The PEI molecules on the surface of MoS_2_ nanosheets in the MoS_2_–polymer composites serve as an interconnected polymer chain network to stabilize the shape and structure of the aerogel-like MoS_2_–polymer composites. After carbonization, these interconnected PEI molecules converted in situ to graphitized carbon layers on the surface of MoS_2_ and interconnected carbon networks, accompanying with the formation of the MoS_2_/NC heteroaerogels.

To systematically investigate the PEI direct-intercalation behaviors, X-ray diffraction (XRD) characterization was applied to the pre-intercalated MoS_2_ nanosheets and MoS_2_–PEI composite, as well as their corresponding annealed MoS_2_ nanosheets and carbonized products of the MoS_2_/NC heteroaerogel (Fig. 2f, g). The pre-intercalated MoS_2_ and the MoS_2_–PEI composite show typical XRD diffraction peaks at 33°, 44°, and 59° assigned to the (101), (103), and (110) planes of the hexagonal-phase MoS_2_ (JCPDS No. 89-5112), respectively. However, the peaks corresponding to (002) plane for the two samples emerge both at lower-angle positions relative to that of the standard MoS_2_ (JCPDS No. 89-5112), showing increased interlayer distances of 10.0 and 10.8 Å, respectively (Fig. 2f and inset). In addition, (004) peaks corresponding to half distance of MoS_2_ interlayers also appear for the two samples. The increase in *d*-spacing of (002) plane for the pre-intercalated MoS_2_ and MoS_2_–PEI composite is ascribed to the intercalation of Na^+^/NH_4_^[+22^ and PEI molecules into the MoS_2_ interlayers, respectively. However, after the thermal treatments, the annealed MoS_2_ and the MoS_2_/NC samples obtained from the pre-intercalated MoS_2_ and MoS_2_–PEI composite exhibit remarkable difference in final interlayer distance. For the annealed MoS_2_, the interlayer distance is reduced to 6.2 Å at ~14° (Fig. 2g), corresponding to the standard value of the normal MoS_2_, which indicates that the inserted NH_4_^+^ ions in the layers of pre-intercalated MoS_2_ were released at a higher temperature over 200 °C^38,39^. Nevertheless, for the MoS_2_/NC heteroaerogel, the interlayer distance of MoS_2_ was reduced to 9.8 Å (Fig. 2g), a value according with that of graphene-inserted MoS_2_ nanosheets^30^, confirming the existence of a graphene-like carbon layer in the interlayers of MoS_2_ induced by the in situ carbonization of PEI. Raman spectroscopy tests also confirmed the formation of the MoS_2_/NC heteroaerogel, as shown in Supplementary Fig. 4. It can be seen that Raman spectrum of the annealed MoS_2_ shows two distinct peaks at 376.5 and 402.6 cm^−1^ ascribed to the typical *E*^1^_2*g*_ (the in-plane displacement of Mo and S atoms), and *A*^1^_*g*_ vibration modes (out-of-plane symmetric displacement of S atom along the *c* axis) of MoS_2_. However, for the MoS_2_/NC heteroaerogel, the two peaks shift to 378.1 and 400.2 cm^−1^, respectively. The blue shift of *E*^1^_2*g*_ peak and the red shift of *A*^1^_*g*_ peak are particular signals induced by the carbon insertion^40,41^. Besides, the other two peaks centered at ~1350.1 and ~1590.9 cm^−1^ corresponding to the typical D and G bands of graphitized carbons are also observed. The mass ratio of MoS_2_ and carbon components was measured to be 74:26 based on thermal gravimetric analysis (TGA) method (Supplementary Fig. 5), during which carbon was completely removed and MoS_2_ was oxidized completely to MoO_3_^12,42^. The carbon content (26%) in the heteroaerogel is contributed by both the carbon in the interlayers and on the surface of MoS_2_ nanosheets.

### Influence of content of polyethyleneimine intercalator

The influence of some key factors such as content and molecular weight (Mw) of PEI on the direct-intercalation behavior and the structure of resulted MoS_2_/NC composites was investigated. Supplementary Fig. 6 shows XRD patterns of MoS_2_–PEI composites after the intercalation of PEI with different mass percentages of 16.7–50 wt%, and their corresponding MoS_2_/NC composites after carbonization. It is noted that the mass percentage of 44.4 wt% is the typical condition for the direct intercalation of PEI and the preparation of the MoS_2_/NC heteroaerogel that have been discussed above. In Supplementary Fig. 6a, the (002) peaks with an expanded *d*-spacing of 10.8 Å are observed in the corresponding XRD patterns of all the four samples of MoS_2_–PEI composites, indicating the effective intercalation of PEI molecules with the various contents. After annealing treatments, the *d*-spacings of (002) peaks for all the samples are reduced, yet to different extents (Supplementary Fig. 6b). The MoS_2_/NC sample derived from a higher PEI content (50 wt%) shows a similar (002) plane *d*-spacing of 9.8 Å to the typical sample (44.4 wt% PEI), indicating the successful insertion of a graphene layer into MoS_2_ nanosheets. However, the high PEI content led to the excessive coating of graphitized carbon on the surface of MoS_2_ nanosheets after carbonization, which may suppress the peak intensity of MoS_2_, as shown in Supplementary Fig. 6b. For the samples derived from lower PEI contents (33.3 and 16.7 wt%), their corresponding *d*-spacings of (002) plane are reduced partially or completely to 6.2 Å, the normal *d*-spacing value of (002) plane of the standard MoS_2_ nanosheets. The results demonstrate that small amounts of PEI may be prone to be expelled out from the interlayers of MoS_2_ nanosheets in the MoS_2_–PEI composites during the carbonization process at high temperatures. Therefore, sufficient PEI is necessary for the preparation of MoS_2_/NC heteroaerogel with expended interlayers of MoS_2_ nanosheets via the direct-intercalation strategy. Supplementary Fig. 7 shows XRD patterns of MoS_2_–PEI composites and their corresponding MoS_2_/NC composites prepared by using PEI with different molecular weights.

### Influence of molecular weight and type of polymer intercalators

Interestingly, it is found that PEI with a wide range of molecular weight from 600 to as high as 70,000, can direct intercalate into the interlayers of MoS_2_ nanosheets (Supplementary Fig. 7a) and exist stably as graphene layers in the MoS_2_ interlayers after carbonization (Supplementary Fig. 7b), leading to the formation of the novel MoS_2_/NC composites. The D, G bands are clearly observed for all the samples, indicating the graphitization of the carbon component in the composites (Supplementary Fig. 8). In contrast, the highest peak intensity ratios, 2.21 and 1.38, of (002)/(101) are obtained for the MoS_2_-PEI composite and its corresponding MoS_2_/NC composite derived from the intercalation of PEI (Mw 600), respectively, indicating the best intercalation effect (Supplementary Fig. 7c). Generally speaking, polymers with a higher molecular weight and a longer chain are more difficult to intercalate into interlayers of MoS_2_ nanosheets. The realization of direct intercalation of PEI may be attributed to the relatively expended interlayer distance (10 Å) of the pre-intercalated MoS_2_ nanosheets and the electrostatic force between the cationic PEI and the negatively charged MoS_2_. To extend the scope of the PDI strategy, polyethylene glycol (PEG) which contains oppositely charged –OH groups was employed and systematically investigated with the influence of its content and molecular weight on the direct intercalation behavior and the formation of MoS_2_/carbon (MoS_2_/C) composites (Supplementary Figs. 9–12). The results indicate that PEG with different molecular weights of 400–20,000 can also serve as an effective intercalator, resulting in a much larger MoS_2_ interlayer distance of 15.6 Å for MoS_2_–PEG composites and a similar interlayer distance of 9.8 Å for the MoS_2_/C composites after carbonization (Supplementary Fig. 11). The detailed information regarding direct intercalation by PEI and PEG and the finally resulted MoS_2_/NC and MoS_2_/C products is summarized in Supplementary Tables 1 and 2.

We noted that the sonication applied in the intercalation process can cause the degradation of polymers, forming lower molecular weight polymers^43–45^ or even small molecules such as oligomers that may intercalate into interlayers of MoS_2_ and result in interlayer expansion. To confirm the intercalation effect is contributed by polymers rather than oligomers generated during sonication of polymers, systematic experiments and measurements using gel permeation chromatography (GPC) and liquid chromatography-mass spectrometry (LC-MS) were conducted (Supplementary Figs. 13–16 and Supplementary Tables 3, 4).

### Molecular dynamics calculations

**Fig. 3:**
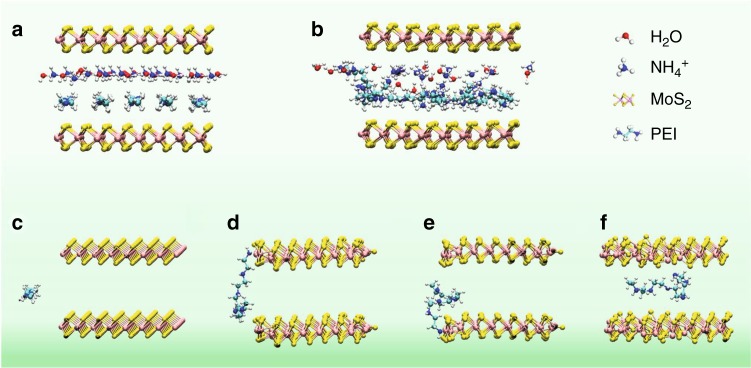
Molecular dynamics calculations. **a**, **b** Schematic illustration shows configurations of PEI-intercalated MoS_2_ with the resulted interlayer distance of 10.8 Å: **a** an initial ordered state; **b** the final relaxed state. **c**–**f** Illustration of PEI molecule intercalation process with the MoS_2_ interlayer distance of 10 Å: **c** 0 ps, **d** 4 ps, **e** 6 ps, **f** 7 ps

The intercalation of 2D layered materials by using ions or small molecule (Li^+^, Na^+^, NH_4_^+^, glucose, etc.) has been well studied^13,23–27^. However, the direct intercalation by polymers has been rarely reported. To explore the configurations of polymer-intercalated MoS_2_ molecular layers and understand how polymers diffused into MoS_2_ interlayers, ab initio molecular dynamics (AIMD) calculations were performed (Supplementary Note 2). Figure 3a, b shows the configurations of PEI-intercalated MoS_2_ with a interlayer distance of 10.8 Å, where a 3D periodic boundary box of 26.58 × 26.58 × 14 Å^3^ is adopted. The calculation results reveal that, 5 PEI molecules together with 18 H_2_O molecules and 17 NH_4_^+^ ions arranging in an initial ordered state and a final relaxed state between the two adjacent monolayers of MoS_2_ nanosheets can both give an interlayer distance of 10.8 Å, agreeing with the experimental value. Similarly, two different configurations with a selected periodic boundary box of 26.58 × 26.58 × 19 Å^3^ containing 5 PEG molecules, 18 H_2_O molecules, and 17 NH_4_^+^ ions are illustrated for PEG-intercalated MoS_2_, showing a calculated interlayer distance of 15.6 Å (Supplementary Fig. 17a, b).

We further performed AIMD to simulate the diffusion process of the polymers intercalating into MoS_2_ interlayers. The simulation was carried out in a periodic boundary box of 23.06 × 40.48 × 13.22 Å^3^. In the *Y* direction, about 20 Å vacuum is added to simulate the edge of MoS_2_. The interlayer distance of MoS_2_ nanosheets is about 10 Å, which is close to our experiment measurement. As shown in Fig. 3c–f and Supplementary Movie 1, a PEI molecule is initially placed outside near the MoS_2_ layers (Fig. 3c, 0 ps). Then, the NH_2_ group in the PEI molecule is attached to the sulfur site on the MoS_2_ edge (Fig. 3d, 4 ps). From 6 ps on, the molecule starts to intercalate into MoS_2_ layers (Fig. 3e). Eventually, the PEI molecule fully enters the interlayer (Fig. 3d, 7 ps). Similarly, for the PEG case, due to the interaction of the OH group and the edge of MoS_2_, the molecule first attaches with edge, then the molecule gradually enters the interlayers of MoS_2_ (Supplementary Fig. 17c–f and Supplementary Movie 2). The intercalation process of the polymers consists of two steps: the first is the interaction between polymer molecules and the edge of MoS_2_ which shorten the distance between the polymer and MoS_2_. And the second, the interaction of the polymer molecule with the MoS_2_ attracts the molecule entering the interlayer. From our static calculations, the binding energy (BE) of a single molecule of PEG and PEI with MoS_2_ is about −1.53 and −1.76 eV, respectively.

**Fig. 4:**
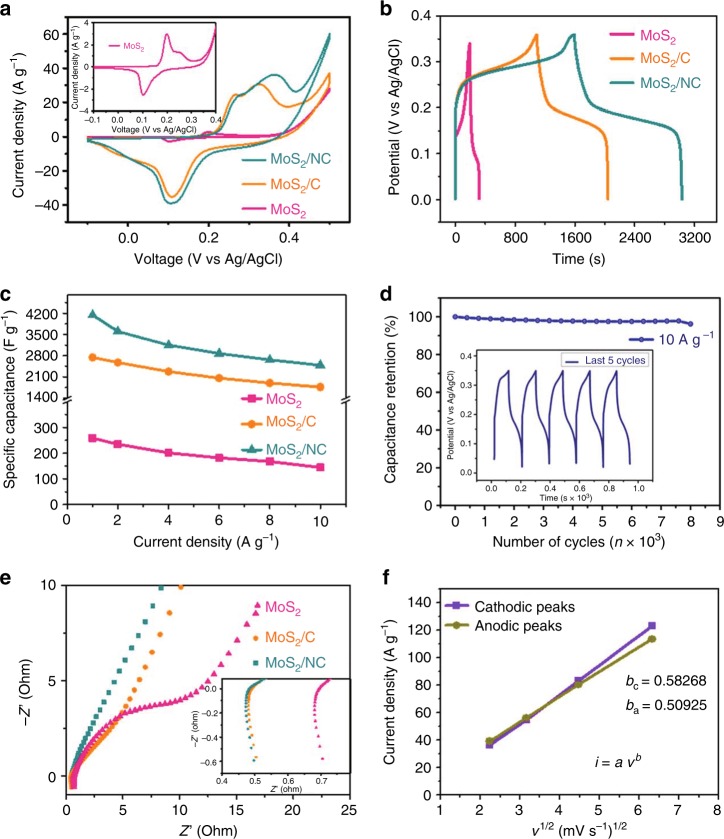
Electrochemical performance. **a**–**c** CV curves at 5 mV s^−1^ (**a**), galvanostatic charging–discharging curves at 1 A g^−1^ (**b**), and specific capacitances at different current densities from 1 to 10 A g^−1^ (**c**) of MoS_2_ nanosheets, and the MoS_2_/C and MoS_2_/NC heteroaerogels. **d** Cycling performance at a current density of 10 A g^−1^ of the MoS_2_/NC heteroaerogel. The inset in (**d**) shows charging–discharging curves of the last 5 cycles. **e** Nyquist plots of the three electrodes. **f** Dependence of the peak current density on square root of sweep rate of the MoS_2_/NC heteroaerogel

It is noted that, the equilibrium interlayer distances induced by the intercalation of PEI and PEG are different (10.8 Å for PEI and 15.6 Å for PEG). We think such difference is attributed to the difference in BE of PEI and PEG to MoS_2_ surfaces and the different behaviors of the two polymers in the water environment. First, PEI has a higher BE than PEG to MoS_2_ surfaces with a stronger interaction, leading to a closer distance between PEI and MoS_2_ layers. Second, the further AIMD simulation indicates that PEI molecules are surrounded by less H_2_O molecules and have a smaller first solvation shell (2.62 Å) than that of PEG (2.75 Å) in aqueous solution (Supplementary Fig. 18). The average distance between PEI molecules and their solvation shell is slightly smaller, which gives rise to a smaller interlayer distance of MoS_2_ nanosheets.

### Electrochemical performance

The electrochemical performance of the MoS_2_-based heteroaerogels as electrodes of supercapacitors was measured in a three-electrode cell in the presence of 6.0 M KOH solution. Figure 4a shows representative cyclic voltammetry (CV) curves of the MoS_2_/NC and MoS_2_/C heteroaerogels as well as the annealed MoS_2_ nanosheets at a sweep rate of 5 mV s^−1^ in the potential range of −0.2 to 0.6 V. The CV curves for all three electrodes display pairs of redox peaks, proving the presence of a reversible Faradic reaction and PC behavior between different valence states of Mo ions (+2 to +6). The peak intensity and curve area for pure MoS_2_ are pretty low (the inset of Fig. 4a), indicating that the capacitance of pure MoS_2_ is negligible. With increasing the scanning rate, the curve shapes for MoS_2_-based heteroaerogels are basically preserved (Supplementary Fig. 19a–c), suggesting fast and stable reversible reactions over the composite electrodes. Figure 4b shows the galvanostatic charge/discharge curves of the three electrodes at 1 A g^−1^, where several sloping plateaus are observed, in good agreement with the CV results. Obviously, the voltage plateaus for the MoS_2_/C and MoS_2_/NC heteroaerogel electrodes, especially the latter, are much more pronounced during both the charging and discharging processes, suggesting a significantly enhanced PC energy storage relative to pure MoS_2_.

An ultrahigh specific capacitance of 4144 F g^−1^ is obtained for the MoS_2_/NC heteroaerogel electrode at a current density 1 A g^−1^, which is much higher than those of the MoS_2_/C heteroaerogel (2576 F g^−1^) and the pure annealed MoS_2_ (259 F g^−1^). This value of specific capacitance also far surpasses those of previously reported high-capacitance electrodes based on metal sulfides, phosphides and oxides, such as NiS nanoframes (2112 F g^−1^ at 1 A g^−1^)^46^, MnCo_2_S_4_ hollow tubular structures (1203 F g^−1^ at 2 A g^−1^)^47^, NiCo_2_S_4_ nanosheets (1231 F g^−1^ at 2 A g^−1^)^48^, Ni_2_P nanosheets arrays (3496 F g^−1^ at 2.5 A g^−1^)^49^, NiO atomic clusters/graphene (3023 F g^−1^ at 1 A g^−1^)^50^, and amorphous Ni(OH)_2_ (3262 F g^−1^ at 5 mV s^−1^), etc^51^. The ultrahigh capacitance of the MoS_2_/NC heteroaerogel should be resulted from its synergistic effect in structure and composition, because the capacitance contributed from the annealed pristine MoS_2_ (Fig. 4a), N-containing carbon (NC) by direct carbonization of PEI and blank electrode of pristine Ni foam (Supplementary Fig. 20) is almost negligible at the same current density. Moreover, the MoS_2_/NC heteroaerogel electrode exhibits higher capacitances of 3550, 3150, 2900, 2670, and 2483 F g^−1^ at current densities of 2, 4, 6, 8, and 10 A g^−1^ than the other two electrodes, respectively, demonstrating an excellent rate capability (Fig. 4c and Supplementary Fig. 19d–f). The MoS_2_/NC heteroaerogel electrode also demonstrates remarkable long-term cycling stability. As shown in Fig. 4d, the electrode shows only an ~8% capacitance decrease after an 8000-cycle charge/discharge test at 10 A g^−1^. The quasi-triangular shape of the discharge/charge curves is still well preserved in the last 5 cycles, indicating the heteroaerogel electrode is stable during the long-term cycling. Such outstanding combined capacitance-rate performances thanks to the PDI strategy which creates efficient pathways for electrons/ions transport and provides more active sites for reversible redox reactions.

### Electrochemical reactions kinetics analysis

As can also be seen in the Nyquist plots, the contact resistance (*R*s) and charge transfer resistance (*R*ct) of the MoS_2_/NC heteroaerogel electrode are both the lowest among the three electrodes (Fig. 4e and the inset), suggesting the excellent conductivity and compatible electrode–electrolyte interface. The Nyquist plots demonstrated a conspicuously decreased *R*ct for the MoS_2_-based heteroaerogels, compared with the pure MoS_2_. The enhanced capability of charge transfer should be ascribed to the graphitized carbon inserting into MoS_2_ interlayers and coating on the surfaces of MoS_2_ nanosheets, which ensures the effective electron transfer between nanosheets in the composite heteroaerogels during capacitive process.

Kinetics analysis of the MoS_2_/NC heteroaerogel electrode was conducted based on their CV curves shown in Supplementary Fig. 19a. From the plots of peak current density (*I*_p_) versus square root of sweep rate (*ν*^1/2^) from 2 to 40 mV s^−1^ for both cathodic and anodic peaks (Fig. 4f), the current follows well a power-law relationship with the sweep rate (*I*_p_ = *aν*^*b*^). A *b*-value of 0.5 indicates that the current is controlled by semi-infinite linear diffusion, whereas a value of 1 indicates that the current is surface-controlled. Specifically, the *b*-value was calculated to be ~0.58 and ~0.51 from the cathodic peak and anodic peak, respectively. The result indicates that the electrode kinetics under the conditions investigated was both diffusion- and surface-controlled, demonstrating a combined PC energy storage contributed from battery-like ion intercalation reactions and fast surface redox reactions. Since the *b*-values are closer to 0.5, the diffusion-controlled ion intercalation effect may be predominant in the whole PC energy storage process, which is facilitated by interlayer distance expansion of the MoS_2_/NC heteroaerogel that allows efficient ion diffusion into/from the interlayers.

### X-ray photoelectron spectroscopy analyses

**Fig. 5:**
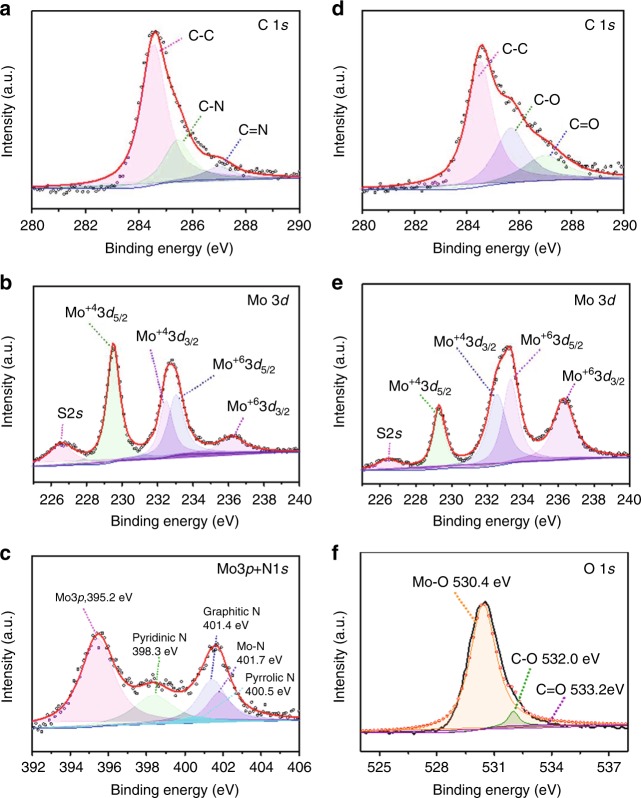
X-ray photoelectron spectroscopy analyses. **a**–**c** High-resolution XPS spectra of C 1*s* (**a**), Mo 3*d* (**b**), and Mo 3*p* + N 1*s* (**c**) of the MoS_2_/NC heteroaerogels. **d**–**f** High-resolution XPS spectra of C 1*s* (**d**), Mo 3*d* (**e**), and O 1*s* (**f**) of the MoS_2_/C heteroaerogels

In order to probe the interfacial interactions between MoS_2_ and carbon-based (C or NC) components, X-ray photoelectron spectroscopy (XPS) examination was conducted for the MoS_2_-based heteroaerogels and the annealed pristine MoS_2_. A survey XPS spectrum of the MoS_2_/NC heteroaerogel provides direct evidence of the presence of C, N, Mo, and S in the heteroaerogel (Supplementary Fig. 21). The C 1*s* spectrum of MoS_2_/NC heteroaerogel exhibits a strong and sharp peak of C–C (284.6 eV) with the weak peaks of C–N (285.5 eV) and C=N (287.0 eV), implying that the graphite carbon is the majority species (Fig. 5a). The two prominent peaks of S 2*p*_1/2_ at 163.2 eV and S 2*p*_3/2_ at 162.2 eV in the core-level S 2*p* XPS spectrum are assigned to the S^2−^ in MoS_2_ of the heteroaerogel (Supplementary Fig. 22), similar to those of the pristine MoS_2_ (Supplementary Fig. 23a). The high-resolution Mo 3*d* XPS of the MoS_2_/NC heteroaerogel reveals that the BEs of Mo 3*d*_3/2_ at 232.4 eV and Mo 3*d*_5/2_ at 229.5 eV are related to the Mo^4+^ ion in the pristine MoS_2_ (Fig. 5b). Notably, compared with pure MoS_2_ (Supplementary Fig. 23b) a new peak at 236.2 eV and a deconvoluted peak at 233.0 eV emerge in the XPS spectrum of Mo 3*d* for the MoS_2_/NC heteroaerogel, corresponding to Mo^6+^ 3*d*_5/2_ and 3*d*_3/2_ respectively (Fig. 5b). The deconvolution of N 1*s* energy level signals for the MoS_2_/NC heteroaerogel reveals the peaks at 398.3, 400.5, and 401.4 eV, which are assigned to pyridinic, pyrrolic, and graphitic N (Fig. 5c), respectively. Although the N 1*s* and Mo 3*p* spectra are partially overlapped, a distinct N 1*s* peak can still be observed at 401.7 eV, assigned to the coordination interactions between the MoS_2_ nanosheets and the N species in the carbon^52,53^. Accordingly, the valence increase of Mo ions is considered as a result of electron transfer from NC component to MoS_2_ via interfacial chemical bonds, i.e., Mo–N bonds between the two components, as evidenced by the Mo 3*p* + N1*s* XPS spectrum in Fig. 5c. For the MoS_2_/C heteroaerogel, in addition to typical C–C bonds as the majority, C–O and C=O bonds derived from PEG intercalator is found existing in the heteroaerogel (Fig. 5d). Interestingly, Mo^6+^ ions are also present, which may be induced by similar electron transfer from Mo to O atoms via interfacial Mo–O bonds between MoS_2_ and carbon components in the composite (Fig. 5e, f)^54^. The strong interactions between MoS_2_ and carbon-based components via the Mo–N or Mo–O bonds can distort the intrinsic Mo–S bonds of MoS_2_, thus leading to a disordered crystal lattice arrangement, as mirrored in Fig. 2b. The above results demonstrate that MoS_2_ nanosheets in the two composites are chemically bonded with the carbon-based components via Mo–N or Mo–O bonds, with increased valence of central Mo ions and efficient across-nanosheet electron transfer pathways through the interfacial bonds.

## Discussion

**Fig. 6:**
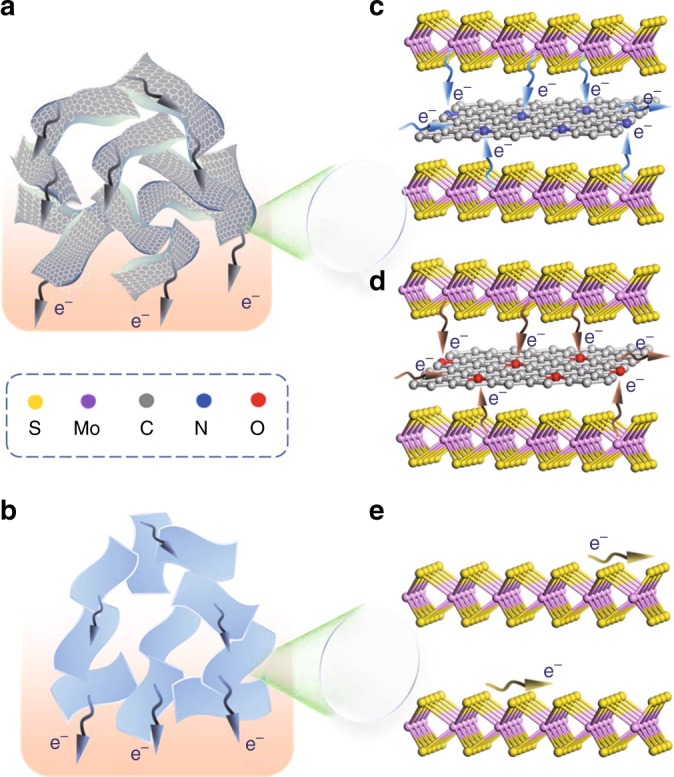
Schematic illustration of different electron transport pathways. **a**, **b** Electron transport pathways between MoS_2_ nanosheets in the MoS_2_-based heteroaerogels (**a**) and pure MoS_2_ powder (**b**). **c**, **d** Electron transport pathways across MoS_2_ monolayers in the MoS_2_/NC heteroaerogel (**c**) and MoS_2_/C heteroaerogel (**d**). **e** Electron transport pathways within MoS_2_ monolayers in pure MoS_2_ powder

Taking all into account, we think the superior PC performance of the MoS_2_-based heteroaerogels, especially the MoS_2_/NC heteroaerogel, is ascribed to their intriguing structural/composition advantages induced by the PDI behavior. First, the 3D heteroaerogels made of the stably stacked MoS_2_ framework and interconnected graphitized carbon network, derived from PEI adsorption on the surface of MoS_2_ nanosheets, offer stable ions diffusion channels and fast electron transport between MoS_2_ nanosheets (Fig. 6a), compared with that of pure MoS_2_ powder (Fig. 6b). Second, expanded interlayers of MoS_2_ nanosheets by the insertion of a graphene-like carbon monolayer derived from PDI expose more accessible active sites for redox reactions and create new pathways for ion/electron transport (Fig. 6c, d), which makes each monolayer of MoS_2_ electrochemically active. For pure MoS_2_, with unexpanded intrinsic interlayers and poor conductivity, electron transfer along the pathways within (002) plane illustrated in Fig. 6e may be very inefficient. In particular, for the MoS_2_/NC heteroaerogel, electron transfer through metallic bond-like interfacial Mo–N bonds within the heteroaerogel is more efficient than that via covalent bond-like Mo–O bonds within the MoS_2_/C heteroaerogel (Fig. 6c, d)^55^. As a consequence, the MoS_2_/NC heteroaerogel electrode demonstrates unprecedent PC energy storage performance.

In summary, the PDI strategy has been proposed for the preparation of interlayer-expanded MoS_2_ nanosheets and MoS_2_/carbon heteroaerogels for ultrahigh-capacitance supercapacitors. The strategy is facile, mild, and efficient, which is applicable to differently charged polymers with a wide range of molecular weight. The direct intercalation behavior of polymers has been investigated systematically by substantial characterizations and molecular dynamic calculations. The resulting MoS_2_/carbon heteroaerogels exhibited outstanding supercapacitor performance such as ultrahigh capacitance, remarkable rate capability, and excellent cycling stability. The superior performance is attributed to the structural and composition advantages of the unique MoS_2_/carbon heteroaerogels with a 3D conductive MoS_2_/C framework, expanded interlayers of MoS_2_ nanosheets, interoverlapped MoS_2_/C layered heterointerface as well as a high content of MoS_2_ (up to 74%) and a high valence (+6) of central Mo ions, which are conducive to not only fast and stable charge transfer/diffusion, but also enhanced ions intercalation pseudocapacitance. The novel PDI strategy may be generally applicable to other 2D layered materials. This study may offer an opportunity for the development of 2D materials-based composites with desirable structure and property for promising energy applications.

## Methods

### Synthesis of pre-intercalated pristine MoS_2_ nanosheets

All the reagents mentioned in the experiment were purchased from Aladdin Biological Technology Co., LTD (China) and used without further purification. The pre-intercalated MoS_2_ nanosheets were synthesized through a simple hydrothermal method. In a typical procedure, sodium molybdate (Na_2_MoO_4_, 0.3 g) and thioacetamide (TAA, C_2_H_5_NS, 0.6 g) were dissolved in DI water (20 mL) mixed with ethylene glycol (10 mL) under continuous stirring for 30 min to form a homogeneous solution. Then, the mixture was transferred into a 100 mL Teflon-lined stainless-steel autoclave, and hydrothermally treated at 220 °C for 24 h. After naturally cooled to room temperature, the resulting black precipitate of pre-intercalated MoS_2_ was collected by filtration and washed with distilled water for several times to remove the residue of reactants. Finally, the obtained MoS_2_ black product was calcined at 300 °C in the flowing argon for 2 h at a heating rate of 2 °C min^−1^ and marked as the annealed pristine MoS_2_.

### Synthesis of MoS_2_/NC and MoS_2_/C heteroaerogels

In a typical synthesis of MoS_2_/NC heteroaerogel, 1.6 g PEI (Mw = 600, Aladdin) and 2.0 g MoS_2_ with a mass ratio of 44 wt% were mixed in a certain amount of water under sonication for 1 h to form a suspension, followed by freeze-drying treatment, resulting in a MoS_2_–PEI composite. Then, the obtained MoS_2_–PEI composite was annealed at 800 °C for 6 h with argon flow, leading to a black product of the MoS_2_/NC heteroaerogel. For the synthesis of MoS_2_/C heteroaerogel, typically, the polymer of PEG (Mw = 400) was employed instead of PEI under otherwise the same conditions. The final composite heteroaerogels prepared by using PEI (Mw = 600) and PEG (Mw = 400) were regarded as the standard samples and were denoted as MoS_2_/NC and MoS_2_/C, respectively. The mass percentage of polymers employed for direct intercalation was 44 wt% if not specified.

### Characterizations

The crystal structure of the samples was characterized by XRD using a D/max2550VB3+/PC X-ray diffractometer with Cu Kα radiation (*λ* = 0.15418 nm) at 40 kV, 100 mA. The 2*θ* degree range used in the measurements was from 5° to 80°. The morphology was observed using a SEM (Hitachi S4800, 3 kV) equipped with an EDS analysis system and a HRTEM (JEM 2011, 200 kV). XPS investigation was conducted in a PHI-5000C ESCA system (PerkinElmer) with Mg Kα radiation (ℏ*ν* = 1253.6 eV). The XPS spectra were measured with a constant analyzer pass energy of 46.95 eV. All BEs were referred to the C 1*s* peak (284.6 eV) arising from surface hydrocarbons (or adventitious hydrocarbon). Raman spectra were recorded by using a spectrophotometer (inVia, Renishaw, Germany) with a 514 nm laser. Thermogravimetric analysis (NETZSCH STA409PC) was carried out from 25 to 700 °C at a heating rate of 10 °C min^−1^ under flowing air. A zeta potential instrument (zetasizer Nano Z) was used for determining the surface charge of the MoS_2_/NC heteroaerogel samples. The specific surface area was measured by the multipoint Brunauere–Emmette–Teller (BET) method at 77.3 K with a Quantachrome NOVA-4200e system. Molecular weight distribution of polymers was studied with GPC using a system from Agilent Technologies 1100. Samples were dissolved in H_2_O. For the measurement, 20 μL of sample was injected. The chromatography was performed at 40 °C using 0.1 mol/L NaNO_3_ as eluent with a flow rate of 0.5 mL/min. Mass spectra were measured on a LC-MS Varian 310 instrument.

### Fabrication and electrochemical test of supercapacitors

All the electrochemical measurements were conducted on a standard three-electrode setup in an electrochemical cell by using a CHI 660E electrochemical workstation (CH Instruments, Inc., Shanghai) in an aqueous KOH electrolyte (6.0 M), where a Pt wire and a saturated Ag/AgCl electrode (filled with saturated KCl with a potential of 0.197 V versus SHE) serve as the counter electrode and the reference electrode, respectively. The working electrode was prepared by mixing active materials, conductive carbon black (Super-P), and polymer binder (polytetrafluoroethylene, PTFE) in a weight ratio of 8:1:1. The mixture was pressed onto a Ni foam that was first treated with a 5% HCl solution, and then was dried at 100 °C for 24 h under vacuum to remove the solvent. The working area of the electrode was set as 1 × 1 cm^2^ and the mass loading of the electrode materials was controlled to be around 2.5 mg. In addition, before each electrochemical test, the electrode was immersed into the electrolyte for 4–5 h.

Cyclic voltammograms (CV), galvanostatic charge/discharge (GCD), and electrochemical impedance spectroscopy (EIS), measurements were performed by using a CHI 660E workstation at ambient temperature. The CVs were recorded from −0.2 to 0.6 V at scan rates of 5–80 mV s^−1^. The GCD measurements were performed at the current densities of 1–40 A g^−1^ with cutoff voltage of 0–0.37 V. EIS was recorded by applying the open-circuit potential with an amplitude of 5 mV over the frequency range from 100 kHz to 0.01 Hz. All potential values for electrochemical measurements were calibrated to the Ag/AgCl reference electrode. Cyclic stability was characterized using galvanostatic charge–discharge measurements over 8000 cycles at a charge–discharge rate of 10 A g^−1^. The specific capacitances of the electrodes are calculated using the following equation^56^:

1 $$C = \frac{{2i_{\it{m}}\mathop {\smallint }\nolimits^ Vdt}}{{V^2|_{V_i}^{V_f}}}$$

where *C* (F g^−1^) is the specific capacitance, *i*_*m*_ = *I*/*m* (A g^−1^) is the current density, where *I* represents the current and *m* represents the active mass of the electrode. $\mathop {\smallint }\nolimits^ Vdt$ represents the integral current area and *V* represents the potential with initial and final values of *V*_*i*_ and *V*_*f*_ respectively.

## Supplementary information

Supplementary information

Supplementary Movie 1

Supplementary Movie 2

## Data Availability

The data that support the findings of this study are available within the paper and its Supplementary Information file, or from the corresponding author on request.
