# Supplementary material for: A polymer-direct-intercalation strategy for MoS2/carbon-derived heteroaerogels with ultrahigh pseudocapacitance
Source: Nat Commun. 2019 Mar 26;10:1372. doi: 10.1038/s41467-019-09384-7 (PMC6435689; doi:10.1038/s41467-019-09384-7)
Supplement: Supplementary file 1 — Supplementary information [file 41467_2019_9384_MOESM1_ESM.pdf]

Supporting Information

A polymer-direct-intercalation strategy for MoS_2/carbon-derived heteroaerogels with ultrahigh pseudocapacitance

Feng et al.

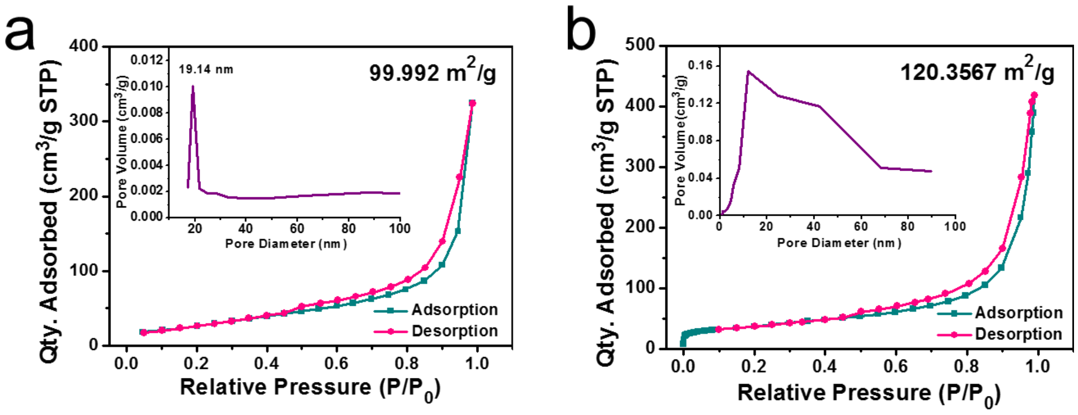

Supplementary Figure 1. Nitrogen adsorption-desorption isotherms of the pre-intercalated MoS_2 nanosheets (**a**) and the MoS_2/NC (PEI, Mw 600) heteroaerogel (**b**).

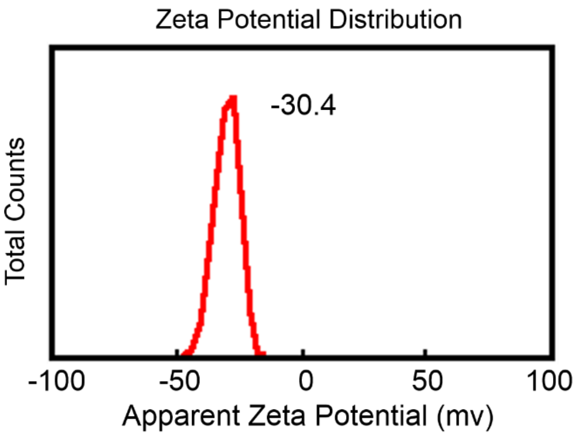

Supplementary Figure 2. Zeta potential curves of the MoS_2 nanosheets in neutral aqueous solution.

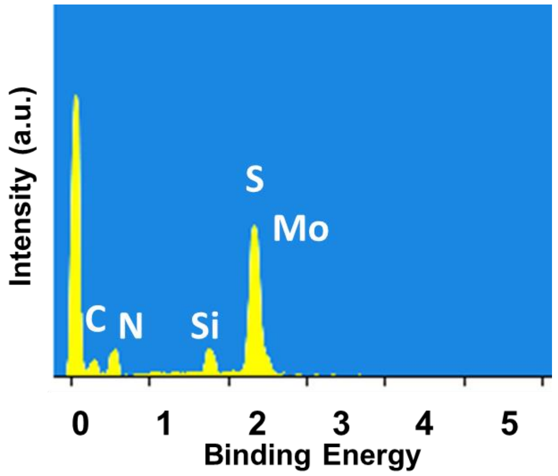

Supplementary Figure 3. EDS results of the MoS_2/NC (PEI, Mw 600) heteroaerogel.

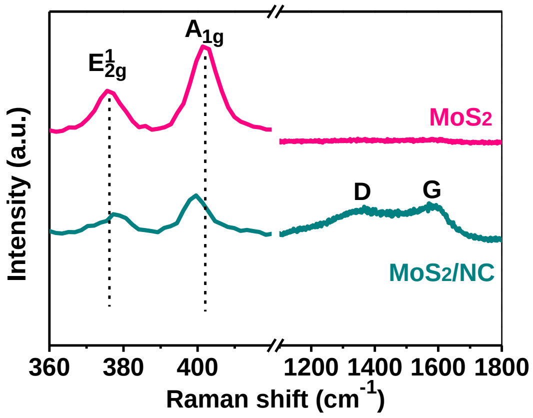

Supplementary Figure 4. Raman spectra of the MoS_2 nanosheets and MoS_2/NC (PEI, Mw 600) heteroaerogel.

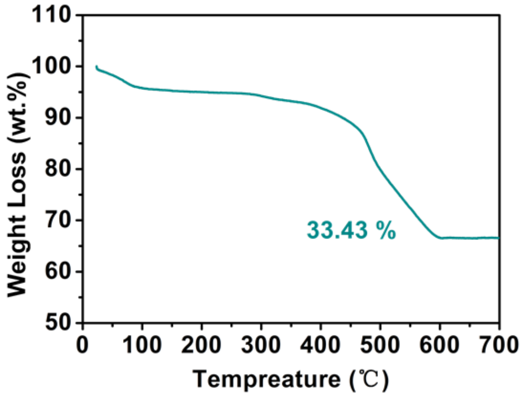

Supplementary Figure 5. TG curves of the MoS_2/NC heteroaerogel with carbon content: 26.10%. During the TG measurement, the carbon component was completely removed by O_2 oxidation and MoS_2 was oxidized completely to MoO_3. Therefore, the MoO_3 was the final residue transformed from MoS_2 in the MoS_2/NC heteroaerogel. Mass of MoS_2 According to the TG data, the mass ratio of the residual MoO_3 is 66.57%, based on which the mass ratio of the residual MoS_2 is calculated to be 74%.

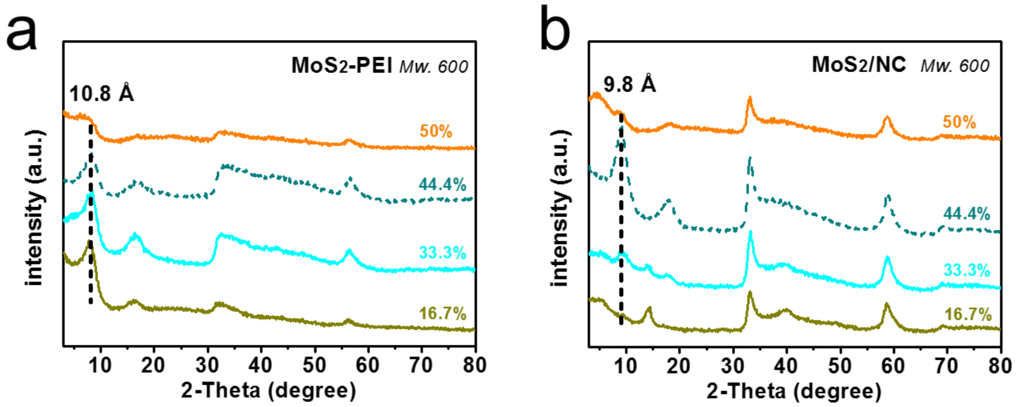

Supplementary Figure 6. XRD patterns of MoS_2-PEI (PEI, Mw = 600) composites after intercalation with different PEI amounts of 16.7%, 33.3%, 44.4% and 50 wt% (**a**), and corresponding MoS_2/NC composites after carbonization (**b**). Mass percentage in this work is calculated relative to total mass of PEI and MoS_2 employed.

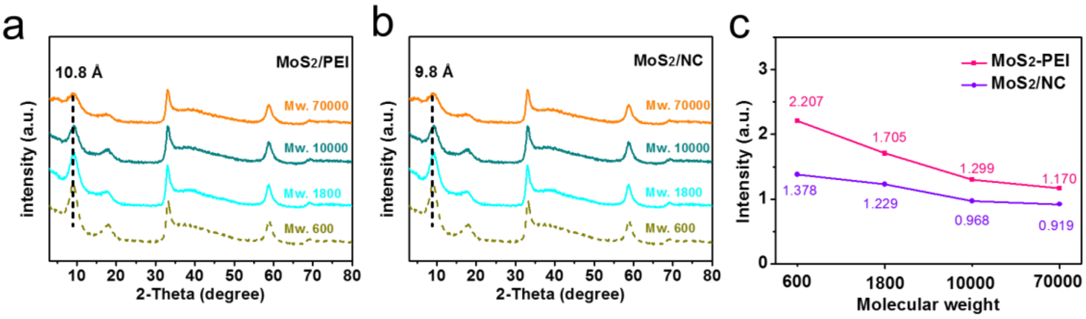

Supplementary Figure 7. XRD patterns of MoS_2-PEI composite (**a**) and MoS_2/NC heteroaerogel (**b**), and the peak intensity ratio of (002) and (101) (**c**) derived from PEI with different molecular weights of 600, 1800, 10000, and 70000.

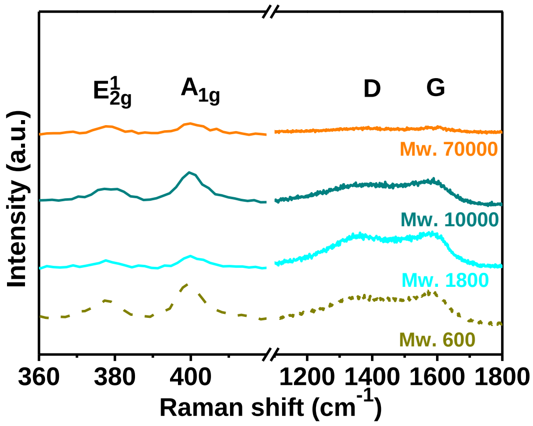

Supplementary Figure 8. Raman spectra of the MoS_2/NC heteroaerogel derived from different PEI molecular weights of 600, 1800, 10000, and 70000.

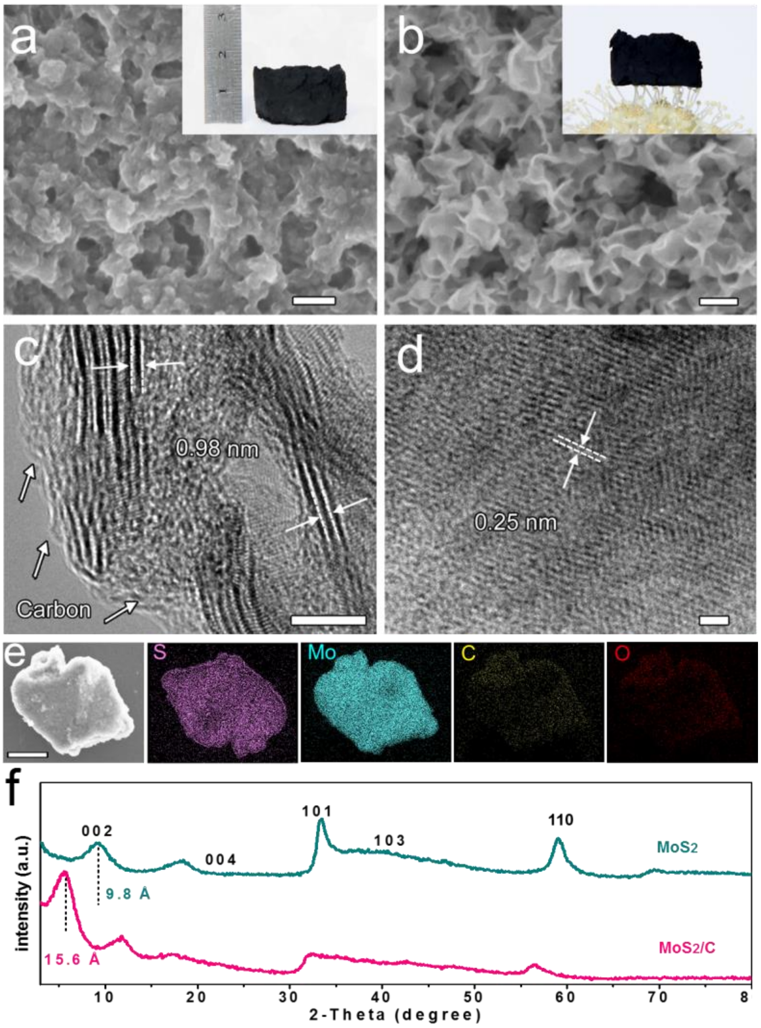

Supplementary Figure 9. **a** SEM image of the MoS_2-PEG (PEG, Mw 400) composite. **b-e** SEM image (**b**), TEM images (**c, d**) and TEM-EDS mapping images (**e**) of the MoS_2/C heteroaerogel. **f** XRD patterns of the MoS_2 nanosheets, and MoS_2/C heteroaerogel. Scale bars: **a,b** 200 nm; **c** 5 nm; **d** 1 nm; **e** 1 μm.

Supplementary Fig. 9a shows the SEM image of the MoS_2-PEG composite after freeze-drying. It can be seen that the surface of MoS_2 nanosheets were coated by PEG intercalator. After carbonization, it is very obvious that the MoS_2 nanosheets hold clear edges, made of intertwined sub-sheets (Supplementary Fig. 9b). The optical photographs of the MoS_2-PEG composite and the MoS_2/C heteaerogel are shown in the insets of Supplementary Fig. 9a, b. The interlayer distance of MoS_2 nanosheets in the MoS_2/C heteroaerogel measured is about 0.98 nm (Supplementary Fig. 9c), which is in agreement with that of the above mentioned MoS_2/C-N heteroaerogel. Furthermore, the atomic arrangement in the (101) plane also exhibits a disordered manner caused by the insertion of a graphitized carbon layer (inset of Supplementary Fig. 9d). The elemental

mapping implies that Mo, S, and C elements are evenly distributed throughout the whole region of the heteroaerogel sample (Supplementary Fig. 9e). Nevertheless, the XRD pattern of the MoS_2-PEG composite presents a new (002) spacing of 15.6 Å (Supplementary Fig. 9f), which is much larger than that (10.8 Å) of the MoS_2-PEI composite. The larger interlayer distance may result from the different functional groups of -OH groups in PEG and the $-NH_2$ groups in PEI which have different solvation behaviors.

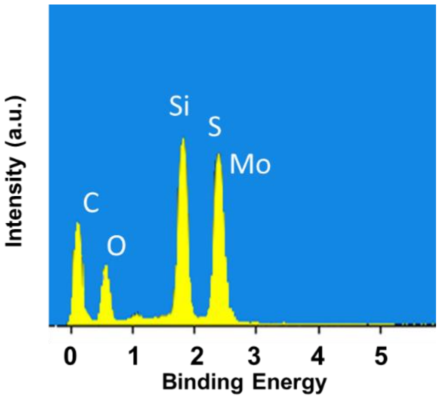

Supplementary Figure 10. EDS result of MoS_2/C (PEG, Mw 400) heteroaerogel.

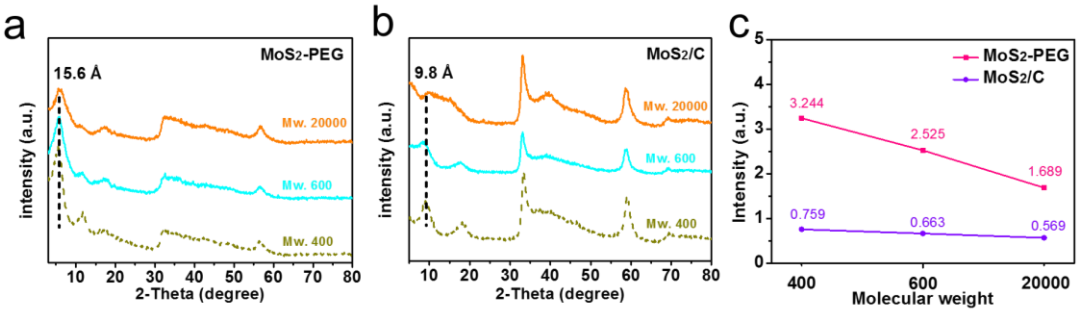

Supplementary Figure 11. XRD patterns of MoS_2-PEG composite (**a**), MoS_2/C heteroaerogel (**b**) and the peak intensity ratio of (002) and (101) (**c**) derived from PEG with different molecular weights of 400, 600 and 20000.

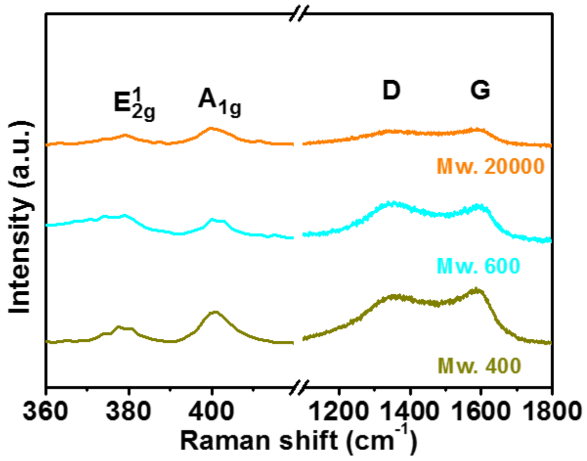

Supplementary Figure 12. Raman spectra of the MoS_2/C heteroaerogel derived from PEG with different molecular weights of 400, 600, and 20000.

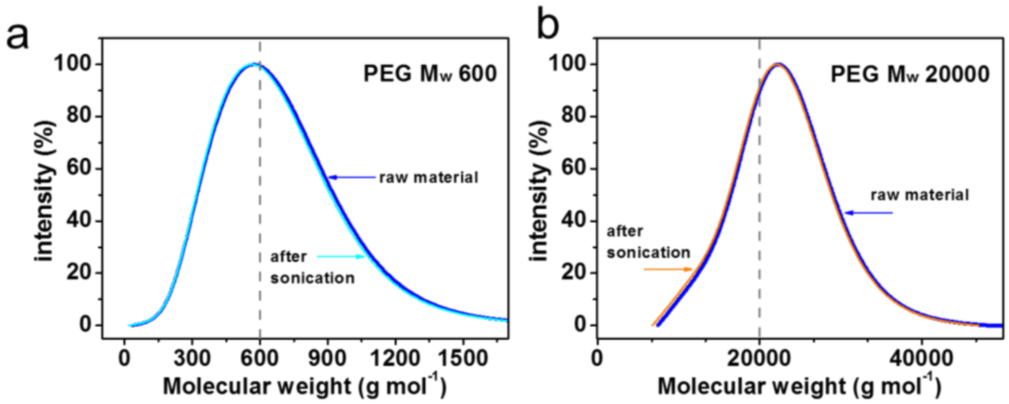

Supplementary Figure 13. GPC analyses of PEG with different molecular weights of 600 (**a**) and 20000 (**b**) without sonication and after sonication for 1h.

Due to the similar structure to standard sample of GPC method, PEG is more suitable for accurate GPC measurement. As shown in Supplementary Fig. 13 and Table 3, PEG with both low (600) and high (20000) molecular weights shows little difference before and after 1h sonication, and no small oligomers are detected.

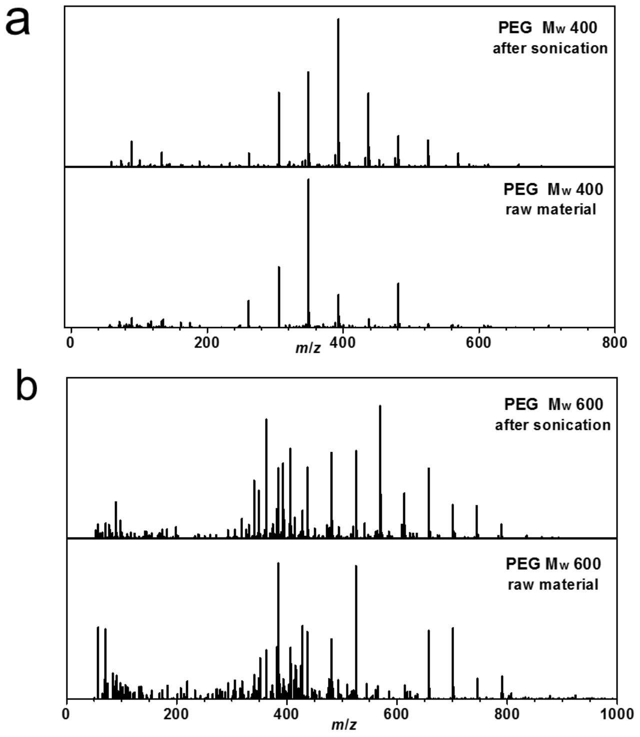

Supplementary Figure 14. LC-MS Spectra of PEG with different molecular weights of 400 (**a**) and 600 (**b**) without sonication and after sonication for 1h.

To precisely determine the existence of small molecules in PEG, mass spectrometry measurement on a liquid chromatography-mass spectrometry (LC-MS) was applied to low-molecular weight PEG (M_w 400 and M_w 600) without and with sonication treatment. It is found that some low-molecular weight oligomers exist in the raw materials of PEG (M_w 400 and 600), and after sonication the oligomers with much lower polymerization degree (n) were nearly not formed (Supplementary Fig. 14 and Supplementary Table 4). The GPC and LC-MS results indicate that sonication can cause the degradation of polymers yet to a low extent.

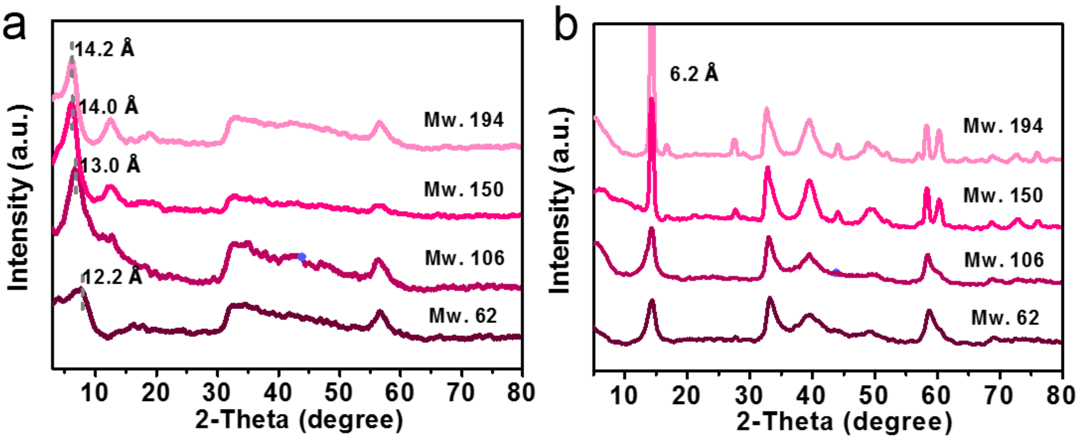

Supplementary Figure 15. XRD patterns of PEG oligomers-intercalated MoS_2 before (**a**) and after annealing treatment (**b**) at 800 °C for 6h.

To further clarify the intercalation behavior of PEG oligomers, a series of oligomers with different degree of polymerization (n = 1, 2, 3, 4) were employed as intercalators for MoS_2 under the same intercalation conditions as the typical. The results in Supplementary Fig. 15a show that all the oligomers can intercalate into the interlayers of MoS_2 and interlayer distance is increased from ~12.2, ~13.0, ~14.0 to ~14.2 Å with increasing the "n" of oligomers. Apparently, all the values of interlayer distance induced by oligomers intercalation are smaller than 15.6 Å corresponding to the case for PEG with higher molecular weights (Mw 400, 600, 20000). Interestingly, after annealing treatment, the expanded interlayer distances of these oligomers-intercalated MoS_2 are reduced back to the normal value of 6.2 Å (Supplementary Fig. 15b), which is the different from the intercalation behavior of polymers demonstrated. The recovery of interlayer distance may be attributed to the low boiling points of these oligomers which are vaporized during the annealing process.

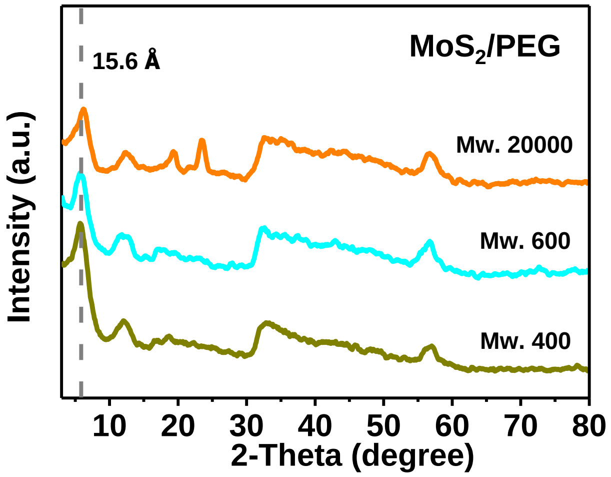

Supplementary Figure 16. XRD patterns of MoS_2-PEG composites formed through intercalation under simply stirring at 300 rpm for 18 h.

To exclude the influence of sonication, the intercalation of the higher-molecular weight PEG (Mw 600, 1800, 20000) under simply stirring without sonication was also conducted. After a longer intercalation process of 18 h, a 15.6 Å large interlayer distance of MoS_2 was obtained as well (Supplementary Fig. 16). Therefore, the sonication seems to offer an extra energy for good dispersion and facilitated intercalation of polymers.

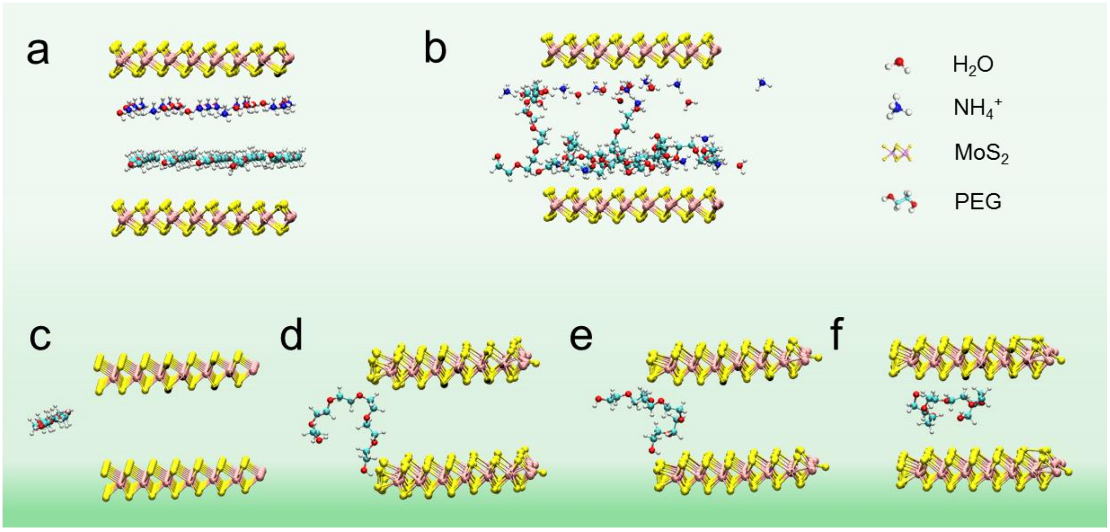

Supplementary Figure 17. **a**, **b** Schematic illustration shows configurations of PEG-intercalated MoS_2 with the resulting interlayer distance of 15.6 Å: **a** an initial ordered state, **b** the final relaxed state. **c-f** Illustration of PEG molecule intercalation process with the MoS_2 interlayer distance of 10 Å: **c** 0ps, **d** 4ps, **e** 6ps, **f** 7ps.

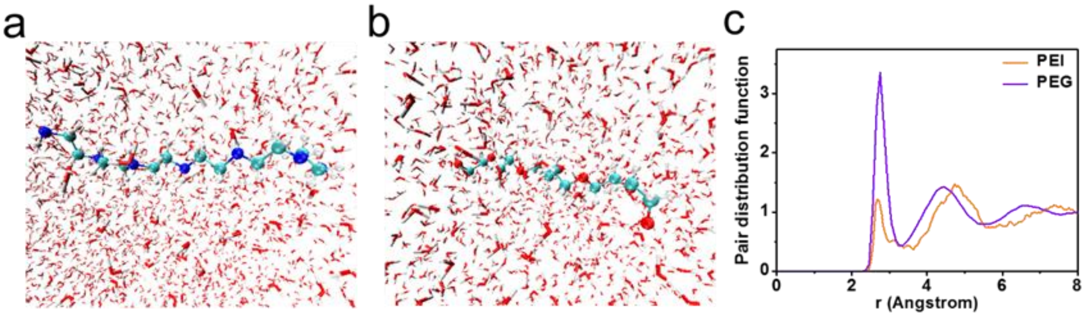

Supplementary Figure 18. **a**, **b** PEI (**a**) and PEG (**b**) in water environment. **c** Pair distribution function of g_{NO} and g_{OO} for PEI and PEG, respectively.

We further carried out molecule dynamics simulation for the PEI and PEG molecules dissociated in water. The simulation was carried out in a three-dimensional boundary box with a volume of $25 \times 25 \times 25$ Å^3. One PEI or PEG molecule is surrounded by 614 water molecules as shown in Supplementary Fig. 14a, b. We first ran NPT ensemble to equilibrate the pressure (1 bar) for 5 ps. Then we changed to NVT for the production run. The sample we used for analysis was 5 ps. We calculated the pair distribution function of g_{NO} (the N atoms in the PEI and O atoms in water) for PEI and g_{OO} (the O atoms in the PEG and O atoms in water) for PEG. The calculated pair distribution function is given in the Supplementary Fig. 14c. As one can see in the figure that the first solvation shell of PEG (2.75 Å) is slightly larger than PEI (2.62 Å). That means the water molecules are slightly closer to PEI molecule than PEG, which is also in agreement with a shorter interlayer distance for PEI intercalation. In addition, the first peak of g_{OO} is more pronounced than the one of g_{NO}, which indicates the PEG molecule are surrounded by more water molecules. To sum up the AIMD simulation for PEI and PEG in water environment, the PEG molecule is surrounded by more water molecule and the average distance between PEG molecule and water solvation shell is slightly larger.

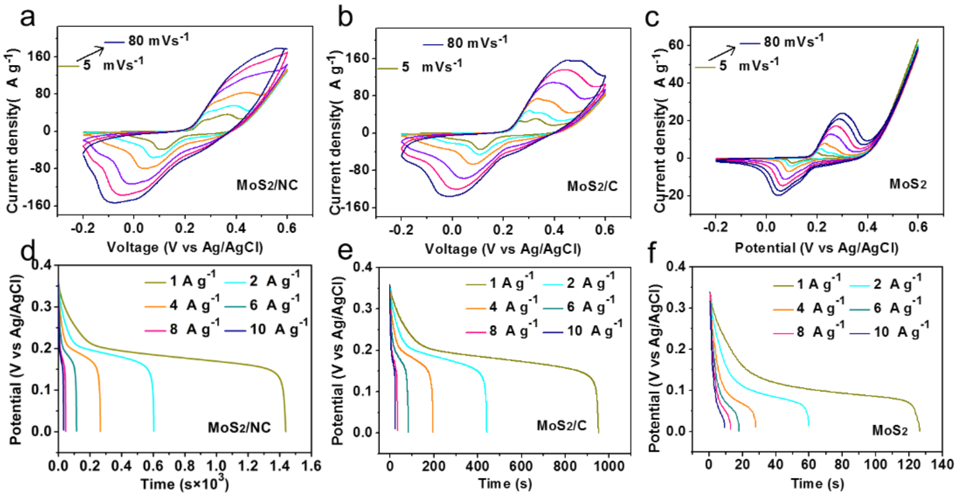

Supplementary Figure 19. CV curves from 5 mV s^{-1} to 80 mV s^{-1} (**a-c**) and Galvanostatic discharging curves at different current density from 1 A g^{-1} to 10 A g^{-1} (**d-f**) of the MoS_2/NC (PEI, Mw 600) heteroaerogel, the MoS_2/C (PEG, Mw 400) heteroaerogel and MoS_2 nanosheets.

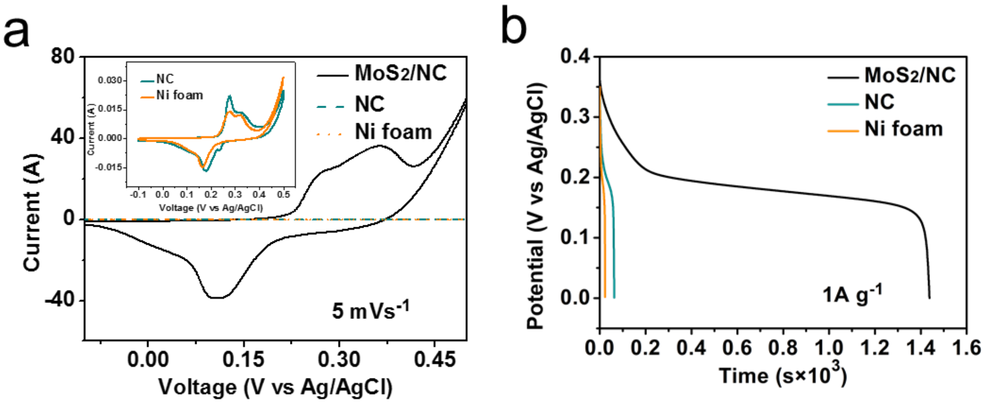

Supplementary Figure 20. **a** CV curves at 5 mV s^{-1} and **b** Galvanostatic discharging curves at 1 A g^{-1} of blank substrate (Ni foam), annealed PEI (NC) and the MoS_2/NC (PEI, Mw 600) heteroaerogel.

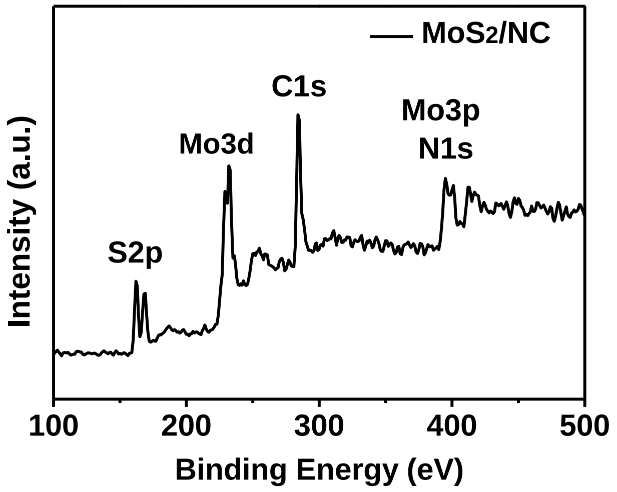

Supplementary Figure 21. XPS survey spectrum of the MoS_2/NC (PEI, Mw 600) heteroaerogel.

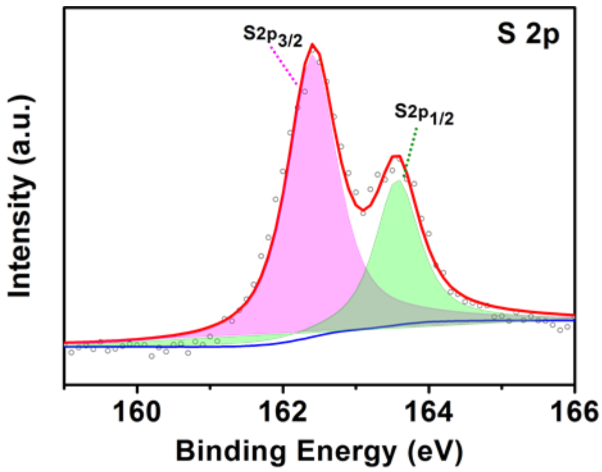

Supplementary Figure 22. The high-resolution XPS spectra of S 2p of the MoS_2/NC (PEI, 600) heteroaerogel.

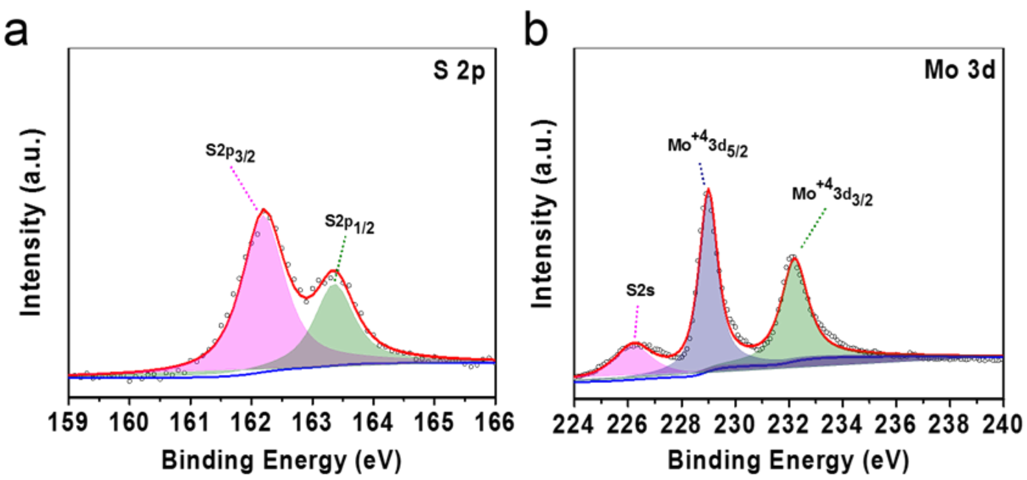

Supplementary Figure 23. The high-resolution XPS spectra of **a** S 2p and **b** Mo 3d of the as-synthesized pristine MoS_2 nanosheets.

Supplementary Table 1. Relationships between the PEI content & carbon content and the (002) interlayer distance of MoS_2 in MoS_2/PEI composites and in MoS_2/NC heteroaerogels.

**MoS_2-PEI composites**	**PEI M_w 600 contents (%)**	**16.7**	**33.3**	**44.4**	**50**
	**Interlayer distance (Å)**	**10.8**			
**MoS_2/NC heteroaerogels**	**C content (%)**	**4.50**	**10.32**	**26.10**	**32.48**
	**Interlayer distance (Å)**	**6.2**	**6.2 & 9.8**	**9.8**	**9.8**

Supplementary Table 2. Relationships between the molecular weight of polymers and structural information of the MoS_2-based heteroaerogels.

	Sample	MoS_2-PEI				MoS_2/NC			
PEI	M_w	600	1800	10000	70000	600	1800	10000	70000
	Interlayer distance (Å)	10.8				9.8			
	I_{002}/I_{101}	2.207	1.705	1.299	1.170	1.378	1.229	0.968	0.919

	Sample	MoS_2-PEG			MoS_2/C		
PEG	M_w	400	600	20000	400	600	2000
	Interlayer distance (Å)	15.6			9.8		
	I_{002}/I_{101}	3.244	2.525	1.689	0.759	0.663	0.569

Supplementary Table 3. Molecular weight of PEG M_w 600 and PEG M_w 20000 without sonication and after sonication measured by the GPC method.

	PEG M_w 600		PEG M_w 20000	
	raw material	after sonication	raw material	after sonication
Mw	595	586	21445	21017
Mn	473	460	19598	19019

Supplementary Table 4. Molecular weight of PEG M_w 400 and PEG M_w 600 without sonication and after sonication measured by the LC-MS method.

n	Mw	PEG 400 Raw material	PEG 400 Sonication	PEG 600 Raw material	PEG 600 Sonication
1	62				
2	106		√		
3	150	√	√		√
4	194		√		√
5	238	√	√		
6	282	√	√	√	√
7	326	√	√	√	√
8	370	√	√	√	√
9	414	√	√	√	√
10	458	√	√	√	√
11	502	√		√	√
12	546	√		√	√
13	590		√		√
14	634		√	√	√
15	678			√	√
16	722			√	
17	766			√	√
18	810				
19	854				
20	898				

Note: “n” in the table represents degree of polymerization for PEG.

Supplementary Note 1. Possible Mechanism forming graphene-like carbon monolayers in the MoS_2 interlayers after carbonization

The formation of graphene-like carbon monolayers in the MoS_2 interlayers after carbonization of the intercalated PEI may be due to the reasons as followings: Firstly, it is speculated that the narrow interlayer distance (1.0 nm) of the interlayer-expended MoS_2 allows only the entrance of single PEI chains along the vertical direction, leading to a single layer of arranged PEI chains confined in the MoS_2 interlayers (as illustrated in Figure 1d); Secondly, in the subsequent carbonization process, subjected to the strong interaction and confinement between the interlayers of MoS_2, the single PEI layers are prone to transform into graphene-like carbon monolayers. It is noted that the formation of carbon monolayers during carbonization is realized by the recognition of carbon atoms derived from carbonized polymers which is actually an atom-concentrating process relative to the intercalated polymer precursors. Consequently, the resulted carbon monolayers may be not totally continuous in the interlayers of MoS_2 nanosheets, but separate into isolated nanodomains. The discontinuous distribution of carbon monolayers offers abundant space for ion diffusion, beneficial for efficient ion transport in the MoS_2 interlayers. With this configuration, the position of the formed carbon monolayers may be not close to the edge of MoS_2 nanosheets. Therefore, it is difficult to directly observe the carbon monolayers in the sandwich-like MoS_2-C-MoS_2 structure, especially when MoS_2-C-MoS_2 heterostructures were highly curved.

Supplementary Note 2. Molecular dynamics calculations

Density function theory (DFT) calculations were performed by using the CP2K package[1]. PBE functional[2] with Grimme D3 correction[3] was used to describe the system. Kohn-Sham DFT has been used as the electronic structure method in the framework of the Gaussian and plane waves method[4,5]. The Goedecker-Teter-Hutter (GTH) pseudopotentials[6,7], DZVP-MOLOPT-GTH basis sets were utilized to describe the molecules[4]. A planewave energy cut-off of 500 Ry has been employed. We perform ab-initio molecular dynamics (AIMD) to study the reasonable configurations of MoS_2 molecular layers after polymer intercalation and the diffusion process of how PEG and PEI intercalate into MoS_2 interlayers. The NVT ensemble has been performed at 300K using Nose-Hoover chain thermostat[8,9].

The binding energy (or adsorption energy) is defined by:

$$E_b = E(mol/MoS_2) - E(MoS_2) - E(mol) \quad (1)$$

Where E_b is the binding energy of the molecule, $E(mol/MoS_2)$ is the total energy of molecule adsorb on MoS_2 surface, $E(MoS_2)$, $E(mol)$ are the energies of surface and molecule, respectively. Due to the high cost for the explicit solvent, we use the implicit solvent model for diffusion process of the polymers intercalating into MoS_2 interlayers[10], which is implemented in CP2K. The water dielectric constant of 78 is used.

Supplementary References

1 Hutter, J., Iannuzzi, M., Schiffmann, F. & VandeVondele, J. cp2k: atomistic simulations of condensed matter systems. *Wiley Interdisciplinary Reviews: Computational Molecular Science* **4**, 15-25, doi:10.1002/wcms.1159 (2014).

2 J. P. Perdew, K. Burke and M. Ernzerhof, *Physical Review Letters*, 1996, 77(18):3865

3 Grimme, S. Semiempirical GGA-type density functional constructed with a long-range dispersion correction. *Journal of computational chemistry* **27**, 1787-1799, doi:10.1002/jcc.20495 (2006).

4 VandeVondele, J. & Hutter, J. Gaussian basis sets for accurate calculations on molecular systems in gas and condensed phases. *J Chem Phys* **127**, 114105, doi:10.1063/1.2770708 (2007).

5 VandeVondele, J. *et al.* Quickstep: Fast and accurate density functional calculations using a mixed Gaussian and plane waves approach. *Computer Physics Communications* **167**, 103-128, doi:10.1016/j.cpc.2004.12.014 (2005).

6 S. Goedecker, M. Teter and J. Hutter, *Physical Review B*, **54**,1703 (1996).

7 C. Hartwigsen, S. Goedecker and J. Hutter, *Physical Review B* **58**, 3641 (1998).

8 Nosé, S. A unified formulation of the constant temperature molecular dynamics methods. *The Journal of Chemical Physics* **81**, 511-519, doi:10.1063/1.447334 (1984).

9 Nosé, S. A molecular dynamics method for simulations in the canonical ensemble. *Molecular Physics* **52**, 255-268, doi:10.1080/00268978400101201 (2006).

10 Bani-Hashemian, M. H., Bruck, S., Luisier, M. & VandeVondele, J. A generalized Poisson solver for first-principles device simulations. *J Chem Phys* **144**, 044113, doi:10.1063/1.4940796 (2016).
